# Supplementary material for: Functional selectivity of insulin receptor revealed by aptamer-trapped receptor structures
Source: Nat Commun. 2022 Oct 30;13:6500. doi: 10.1038/s41467-022-34292-8 (PMC9618554; doi:10.1038/s41467-022-34292-8)
Supplement: Supplementary file 1 — Supplementary Information [file 41467_2022_34292_MOESM1_ESM.pdf]

Supplementary Information

Functional selectivity of insulin receptor revealed by aptamer-trapped receptor structures

Junhong Kim, Na-Oh Yunn, Mangeun Park, Jihan Kim, Seongeun Park, Yoojoong Kim, Jeongeun Noh, Sung Ho Ryu, Yunje Cho

Supplementary Figures

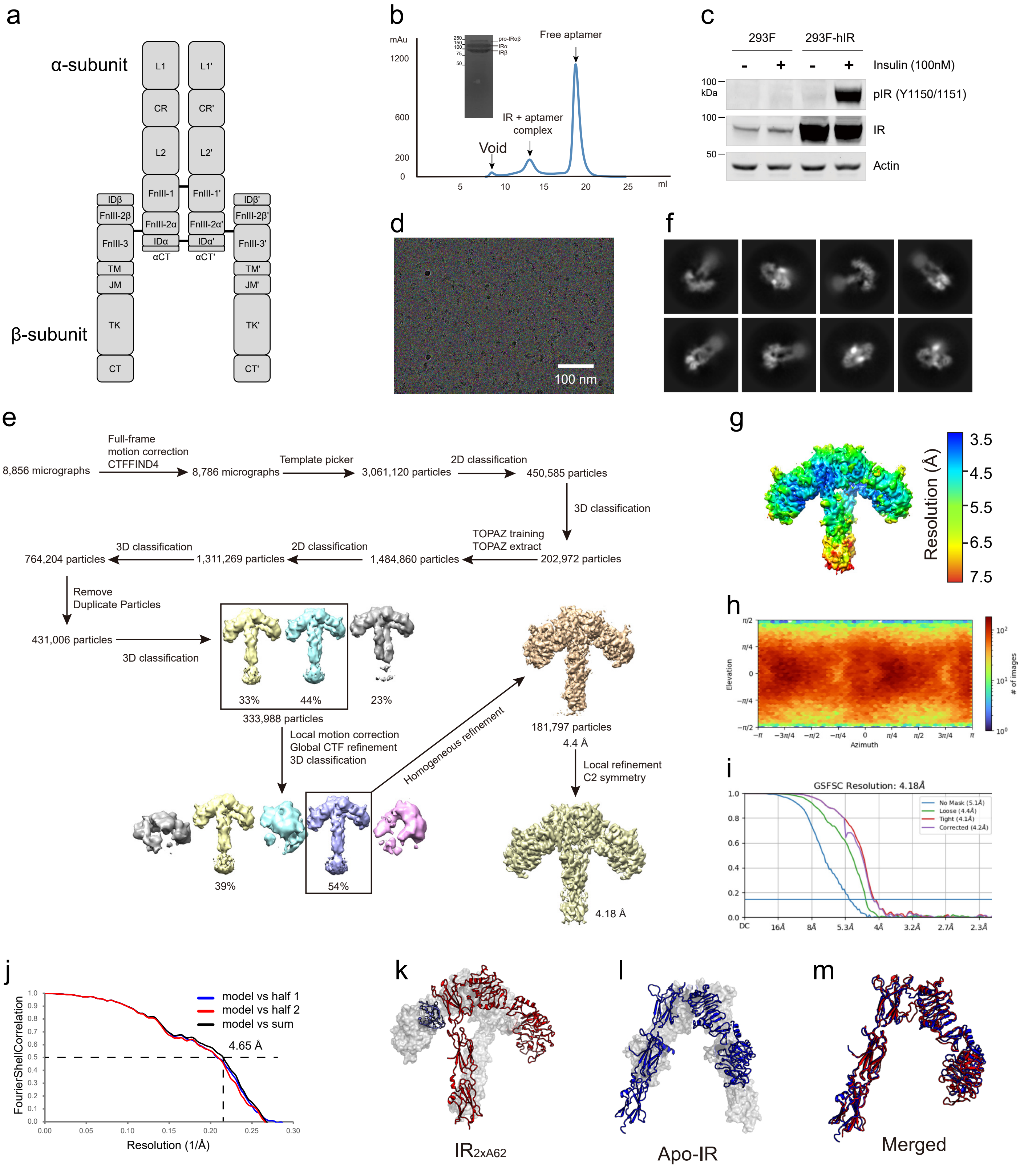

Supplementary Fig. 1. Workflow of cryo-EM processing for the IR$_{2xA62}$ complex, and analysis of the quality of the cryo-EM map. a, Architecture of IR, **b,** A representative size-exclusion chromatography profile of the IR-aptamer complex. **c,** Insulin-induced IR phosphorylation (pY1150/pY1151) in 293F cells or 293F cell lines stably expressing full-length human IR (hIR). Cells were stimulated with 100 nM insulin for 5 min. The data were representative of one experiment. Source data are provided as a Source Data file. **d,** Representative images of raw micrographs of IR$_{2xA62}$ from 8,856 movies in vitrified ice. Data were repeated independently three times with similar results. **e,** Flowchart for single-particle cryo-EM processing. **f,** Representative images of 2D class averages of IR$_{2xA62}$ from reference-free alignment and classification. **g,** Local resolution of the cryo-EM map. **h,** Angular distribution of the final reconstruction of the maps at 4.18 Å. **i,** Gold standard Fourier shell correlation (FSC) curves for refined maps at 4.18 Å. **j,** Map-to-model FSC curves between the refined structure and the maps. **k,** Front view of the arrowhead-shaped IR$_{2xA62}$ complex. One protomer is shown in surface representation and the other protomer is shown as ribbons. **l,** Front view of the inverted V-shaped ligand-free IR (PDB: 4ZXB)[1]. **m,** Superposition of the protomers shown in **1k** (red) and **1l** (blue).

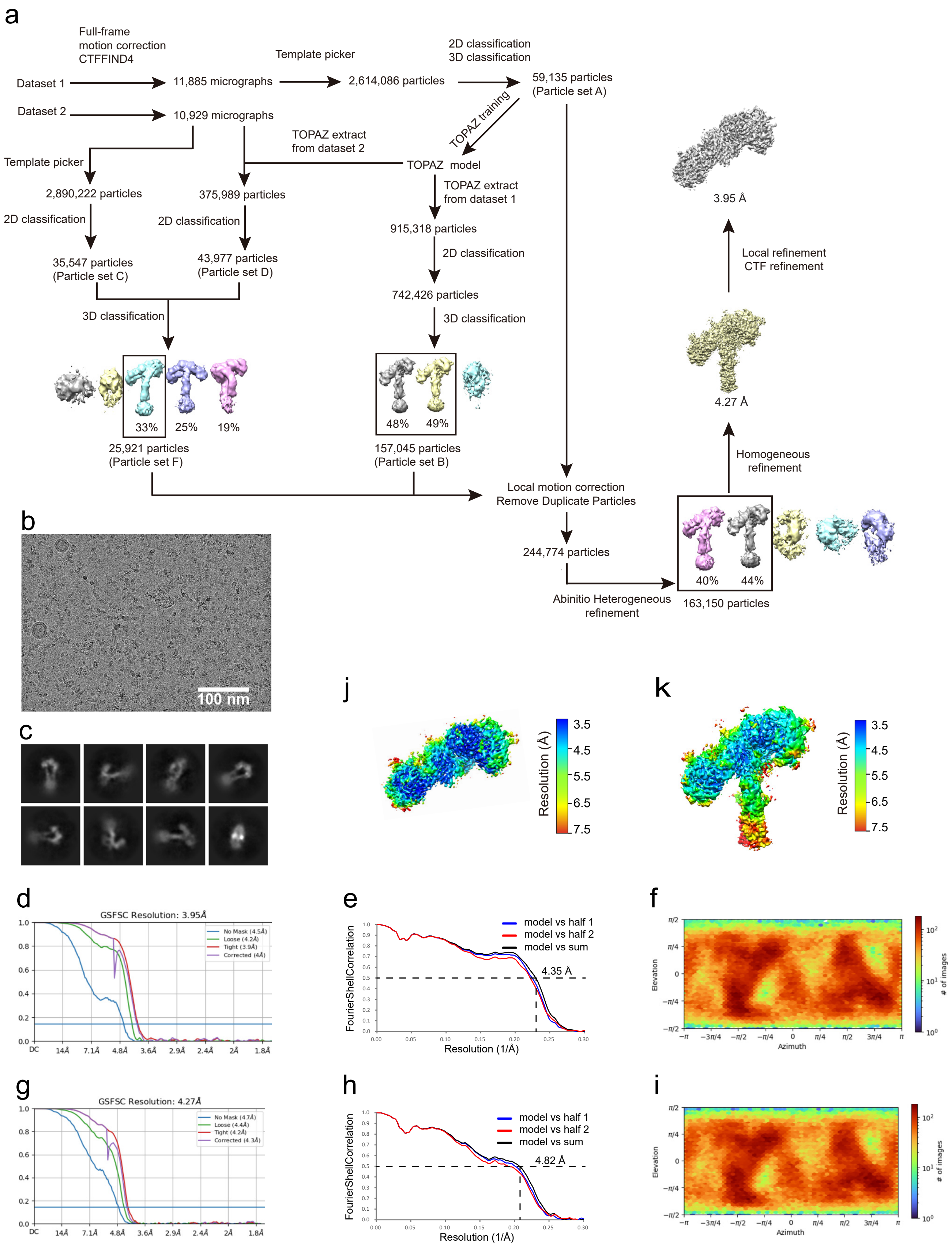

Supplementary Fig. 2. Workflow of cryo-EM processing for the $IR_{A62+Ins}$ complex and analysis of the quality of the cryo-EM map. **a,** Flowchart for single-particle cryo-EM processing. **b,** Representative images of raw micrographs of IR_{2xA62} from 24,323 movies in vitrified ice. Data were repeated independently three times with similar results. **c,** Representative images of 2D class averages of $IR_{A62+Ins}$ from reference-free alignment and classification. **d,** Gold standard FSC curves for locally refined maps at 3.95 Å. **e,** Map-to-model FSC curves between the refined structure and maps of the upper part of the locally refined $IR_{A62+Ins}$ complex. **f,** Angular distribution of the final reconstruction of the upper part of the locally refined maps at 3.95 Å. **g,** Gold standard FSC curves for the overall $IR_{A62+Ins}$ complex maps at 4.27 Å. **h,** Map-to-model FSC curves between the refined structure and maps of the overall $IR_{A62+Ins}$ complex. **i,** Angular distribution of the final reconstruction of the overall maps at 4.27 Å. **j,** Local resolution of the upper part of the locally refined $IR_{A62+Ins}$ complex cryo-EM map. **k,** Local resolution of the overall $IR_{A62+Ins}$ complex cryo-EM map.

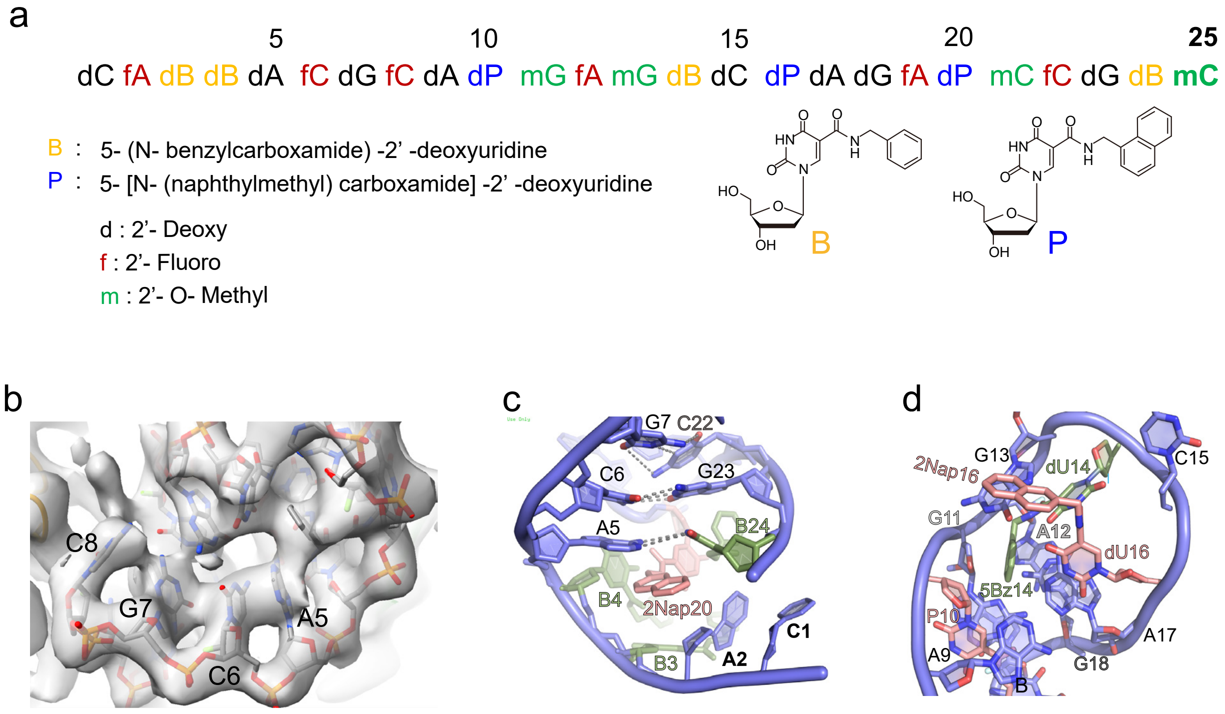

Supplementary Fig. 3. Structural and schematic representations of the A62 aptamer. a, Sequence of the A62 aptamer. Structures of the chemically modified nucleotides are shown below. **b,** A representative part of the cryo-EM map of A62 bound to IR. **c, d,** Two representative local structures of A62.

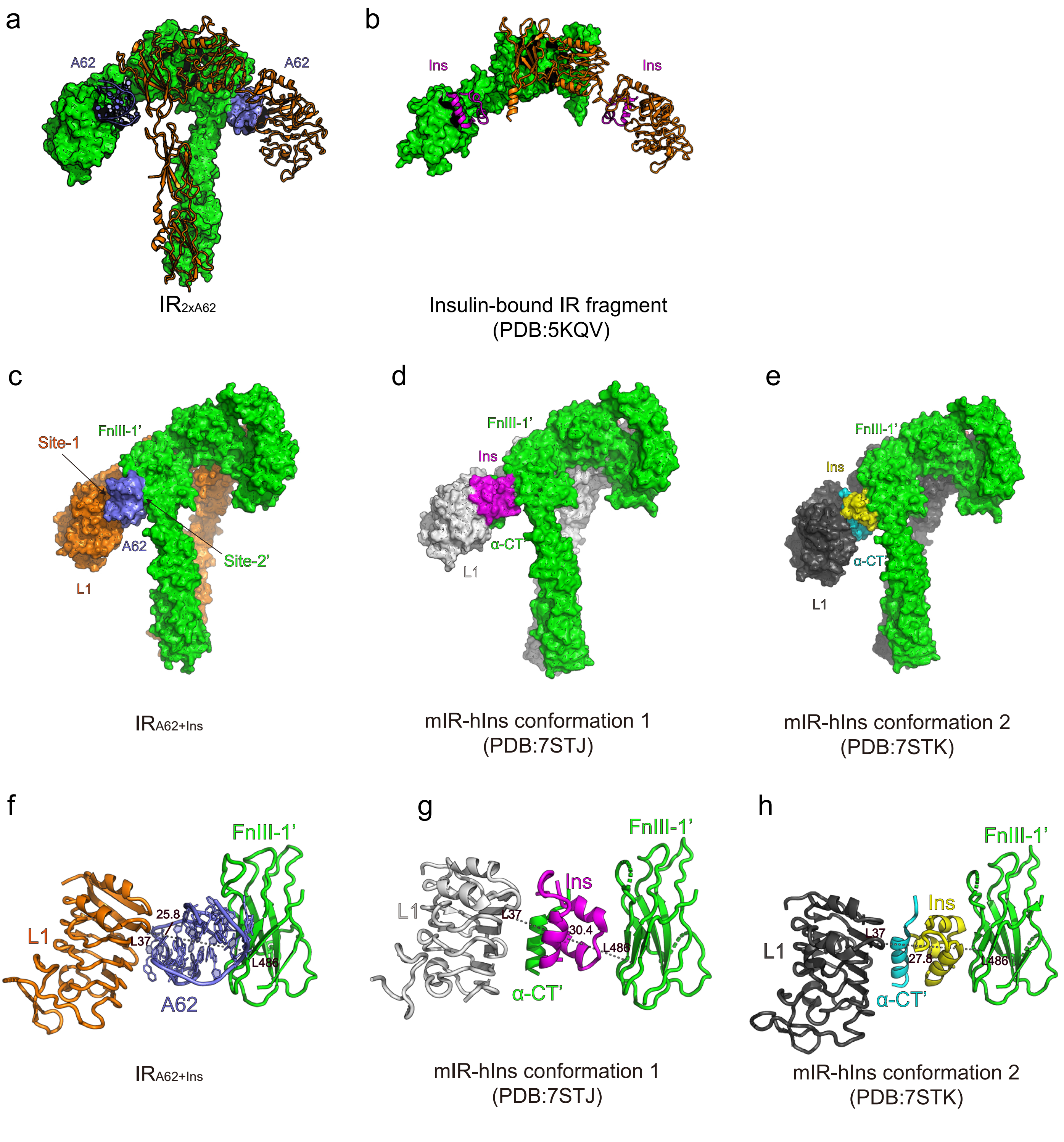

Supplementary Fig. 4. A62 binding mimics site-1/site-2 insulin coordination. Structural comparison of **a,** the head domain of IR_{2xA62} and **b,** the insulin-bound IR fragment (L1, CR, L2, FnIII-1 and αCT, 5KQV)[2]. Structure of the complexed Fab is omitted for clarity. In contrast to A62, insulin only interacts with L1 in the insulin-bound IR fragment. Structural comparison of tilted T shaped IR conformations induced by **c,** one A62 and one insulin ($IR_{A62+Ins}$), and **d, e,** two asymmetrically bound insulins. **f–h,** Close-up view of the A62 bridge or insulin bridge across L1 and FnIII-1' in Supplementary Fig. 4c–e. Dotted line with a number indicates distance between L37 (L1) and L486 (FnIII-1').

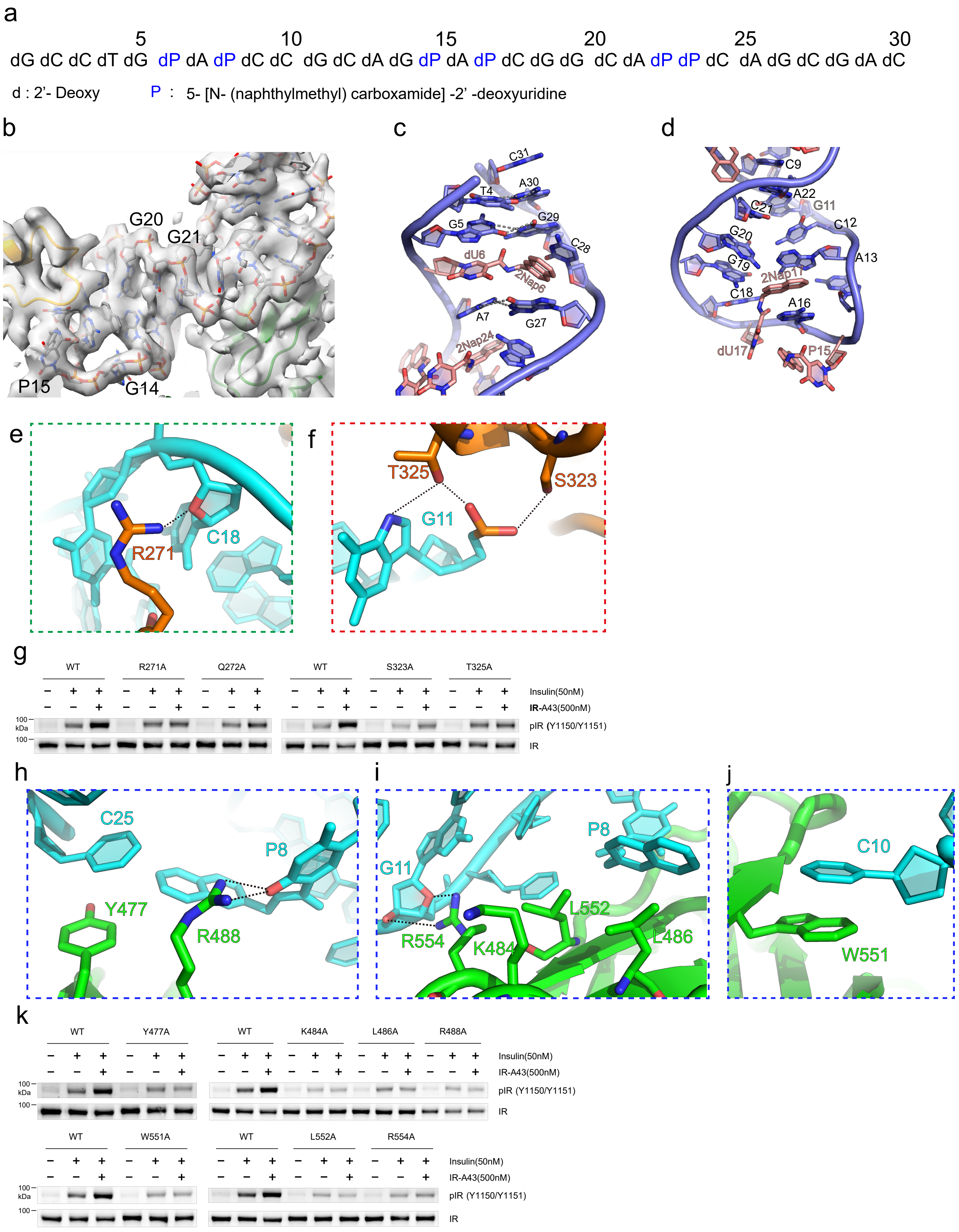

Supplementary Fig. 6. Structural and schematic representations of the A43 aptamer. a, Sequence of the A43 aptamer. **b,** A representative part of the cryo-EM map for A43. **c, d,** Two representative local structures of A43. **e, f,** Close-up view of A43 binding to **(e)** CR and **(f)** L2 domains (green and red boxes in Fig. 3c). **h–j,** Close-up view of A43 binding to FnIII-1' domains (blue box in Fig. 3c). **g, k,** Insulin-induced IR phosphorylation (pY1150/pY1151) in CHO-K1 cells expressing WT IR or indicated point mutants predicted to disrupt A43 binding to **(g)** CR, L2, and **(k)** FnIII-1' domains. Cells were stimulated with 50 nM insulin for 5 min in the presence or absence of 500 nM A43. The data were representative of three independent experiments. Source data are provided as a Source Data file.

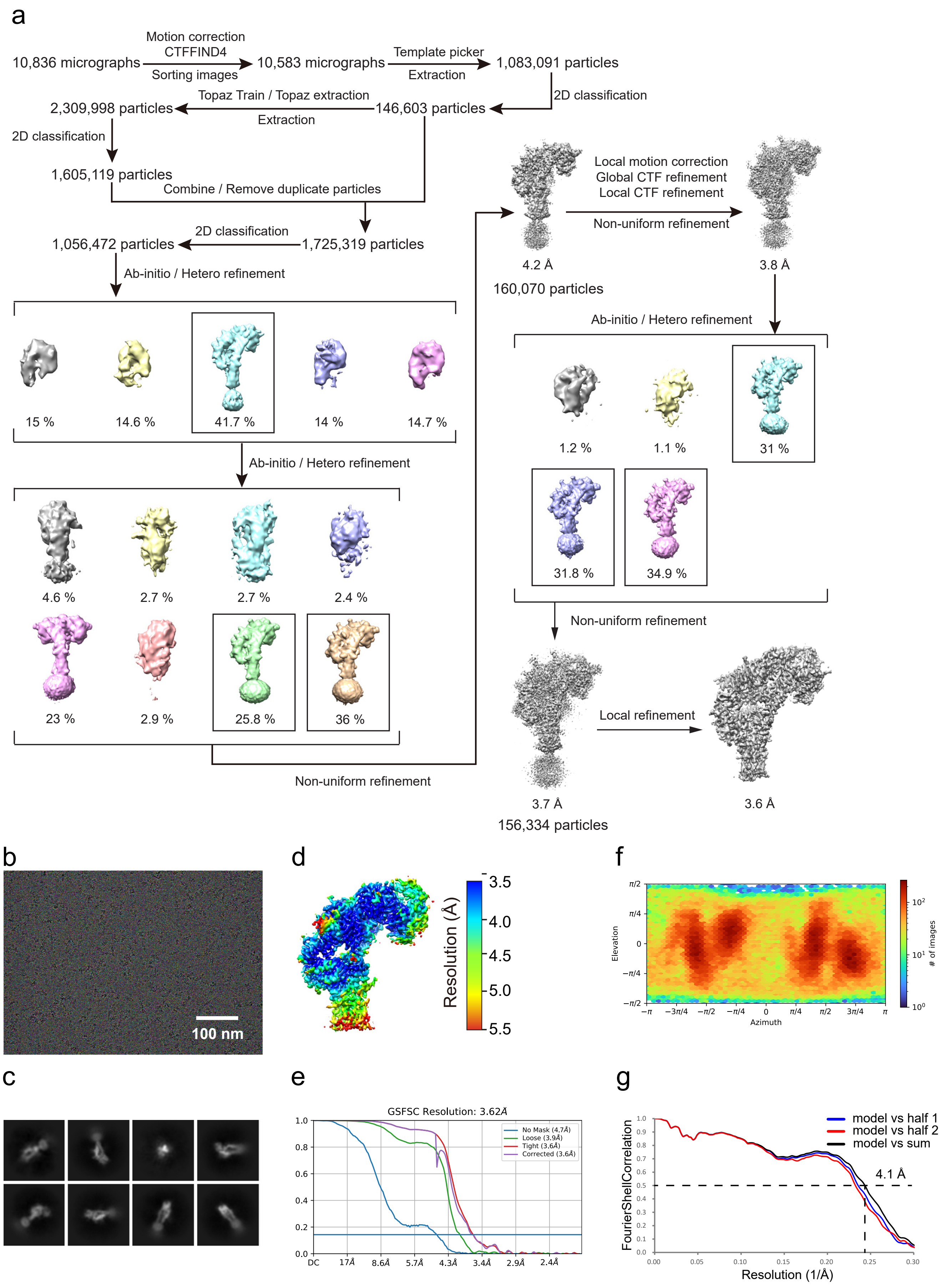

Supplementary Fig. 5. Workflow of cryo-EM processing for the $IR_{A43+Ins}$ complex and analysis of the quality of the cryo-EM map. a, Flowchart for single-particle cryo-EM processing. **b,** Representative images of raw micrographs of $IR_{A43+Ins}$ from 10,836 movies in vitrified ice. **c,** Representative images of 2D class averages of $IR_{A43+Ins}$ from reference-free alignment and classification. **d,** Local resolution of the cryo-EM map. **e,** Gold standard FSC curves for globally refined maps at 3.62 Å. **f,** Angular distribution of the final reconstruction of the maps at 3.62 Å. **g,** Map-to-model FSC curves between the refined structure and the maps.

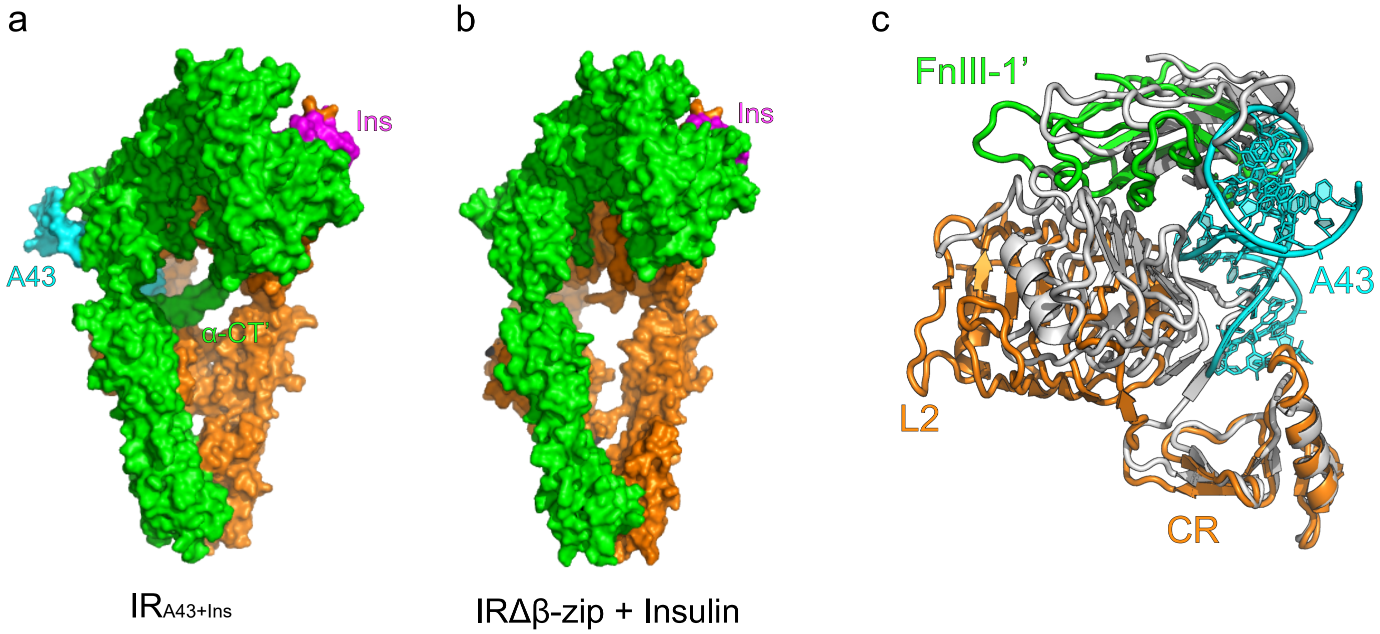

Supplementary Fig. 7. Comparison of the $IR_{A43+Ins}$ complex with single insulin-bound IR. a, Surface representation of the $IR_{A43+Ins}$ structure. Each protomer is shown in orange or green. A43 is colored cyan and insulin is colored magenta. **b,** Surface representation of the single insulin-bound IR structure (PDB: 6HN5, 6HN4)[3]. Each protomer is shown in orange or green. Insulin is colored magenta. **c,** Structural comparison of CR, L2, and FnIII-1' domains between apo-IR (PDB: 4ZXB)[1] and $IR_{A43+Ins}$ by aligning the CR domain. CR, L2, and FnIII-1' domains of apo-IR are colored gray. Domains from $IR_{A43+Ins}$ are colored orange (CR and L2) and green (FnIII-1'). A43 is colored cyan.

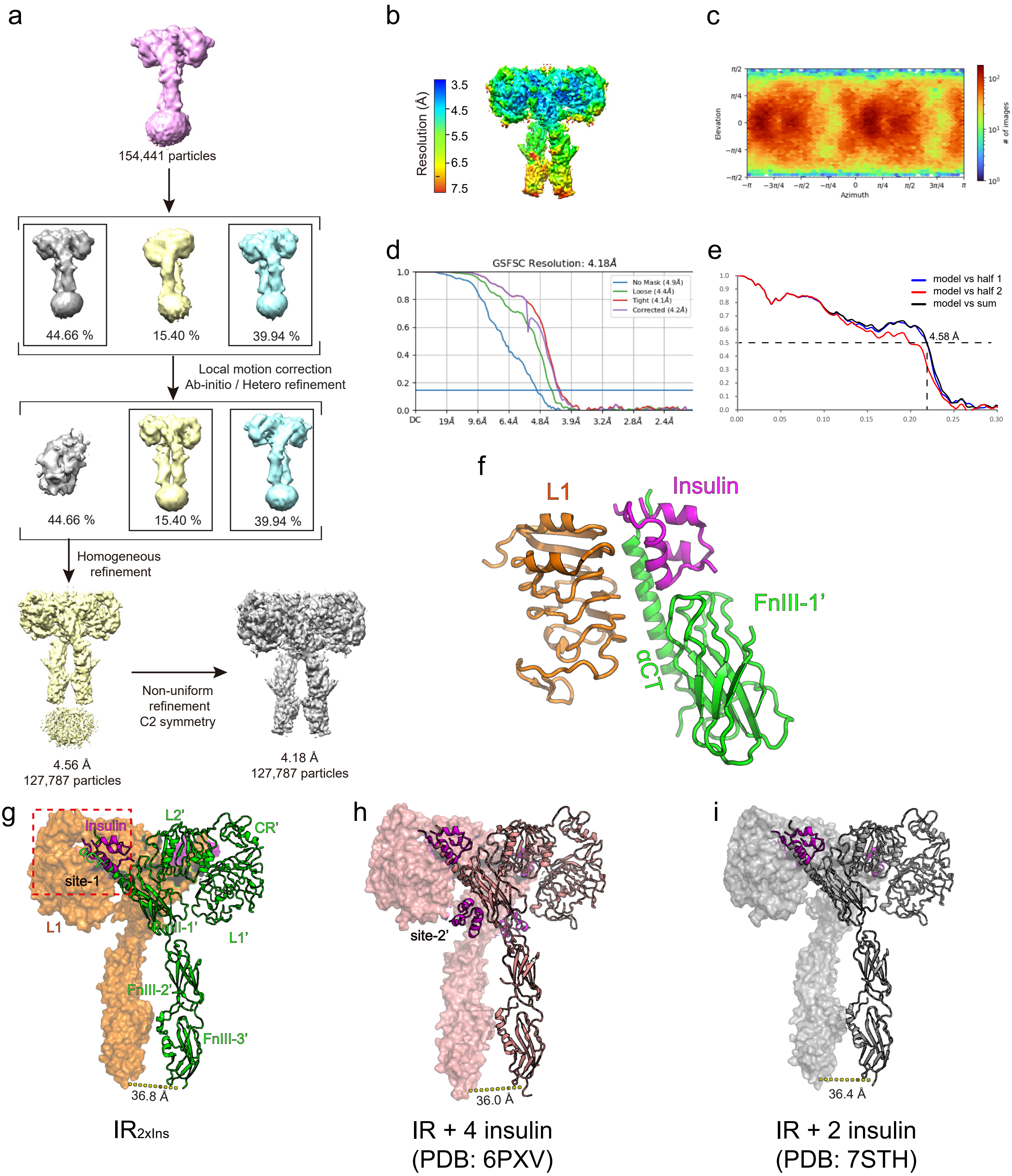

Supplementary Fig. 8. Workflow of cryo-EM processing for the IR$_{2xIns}$ complex, analysis of the quality of the cryo-EM map and comparison with other T-shaped IR structures. a, Flowchart for single-particle cryo-EM processing. **b,** Local resolution of the cryo-EM map. **c,** Angular distribution of the final reconstruction of the maps at 4.18 Å. **d,** Gold standard FSC curves for globally refined maps at 4.18 Å. **e,** Map-to-model FSC curves between the refined structure and the maps. **f,** Close-up view of the insulin binding site (site-1, red box in Supplementary Fig. 8 g). **g,** Structure of IR$_{2xIns}$, with one protomer (orange) shown in surface representation and another protomer (green) in ribbon representation. Insulin molecules are colored magenta. Structural comparison with other T-shaped IR complexed with **h,** four insulin molecules (6PXV)[4] and **i,** two insulin molecules (7STH)[5]. Although not illustrated here, the IR ectodomain with 4 insulin molecules (PDB: 6SOF)[6] also shows similar conformation.

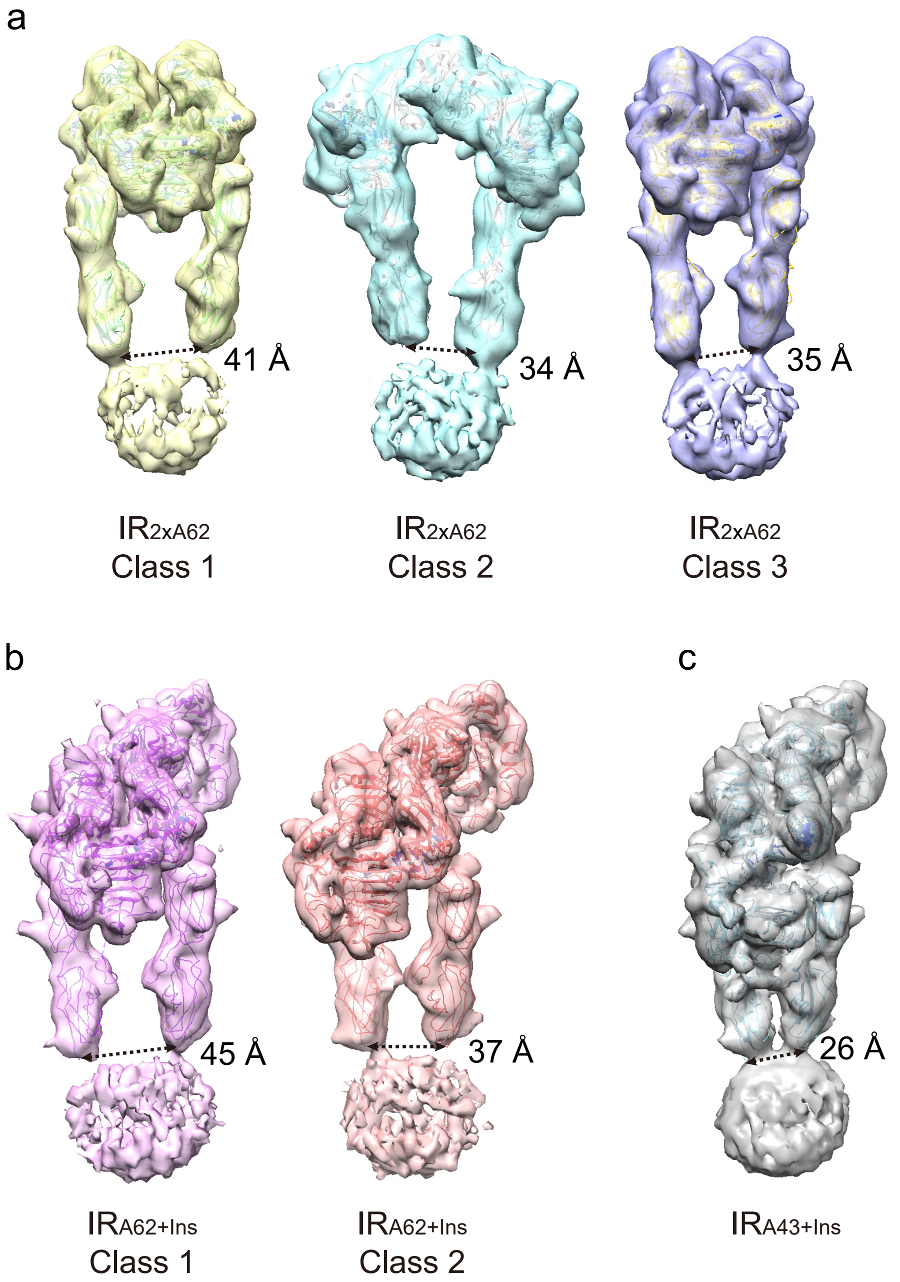

Supplementary Fig. 9. Comparison of distances between FnIII-3 and FnIII-3' from IR_{2xA62}, $IR_{A62+Ins}$, and $IR_{A43+Ins}$ structures. For accurate comparison, after docking the model on the map of all classes from processing steps, the distance of Asp907 between FnIII-3 and FnIII-3' was measured and displayed. **a,** IR_{2xA62} is divided into three classes; class 1 is colored yellow, class 2 is colored cyan, and class 3 is colored purple. **b,** $IR_{A62+Ins}$ is divided into two classes; class 1 is colored magenta and class 2 is colored pink. **c,** $IR_{A43+Ins}$ colored gray.

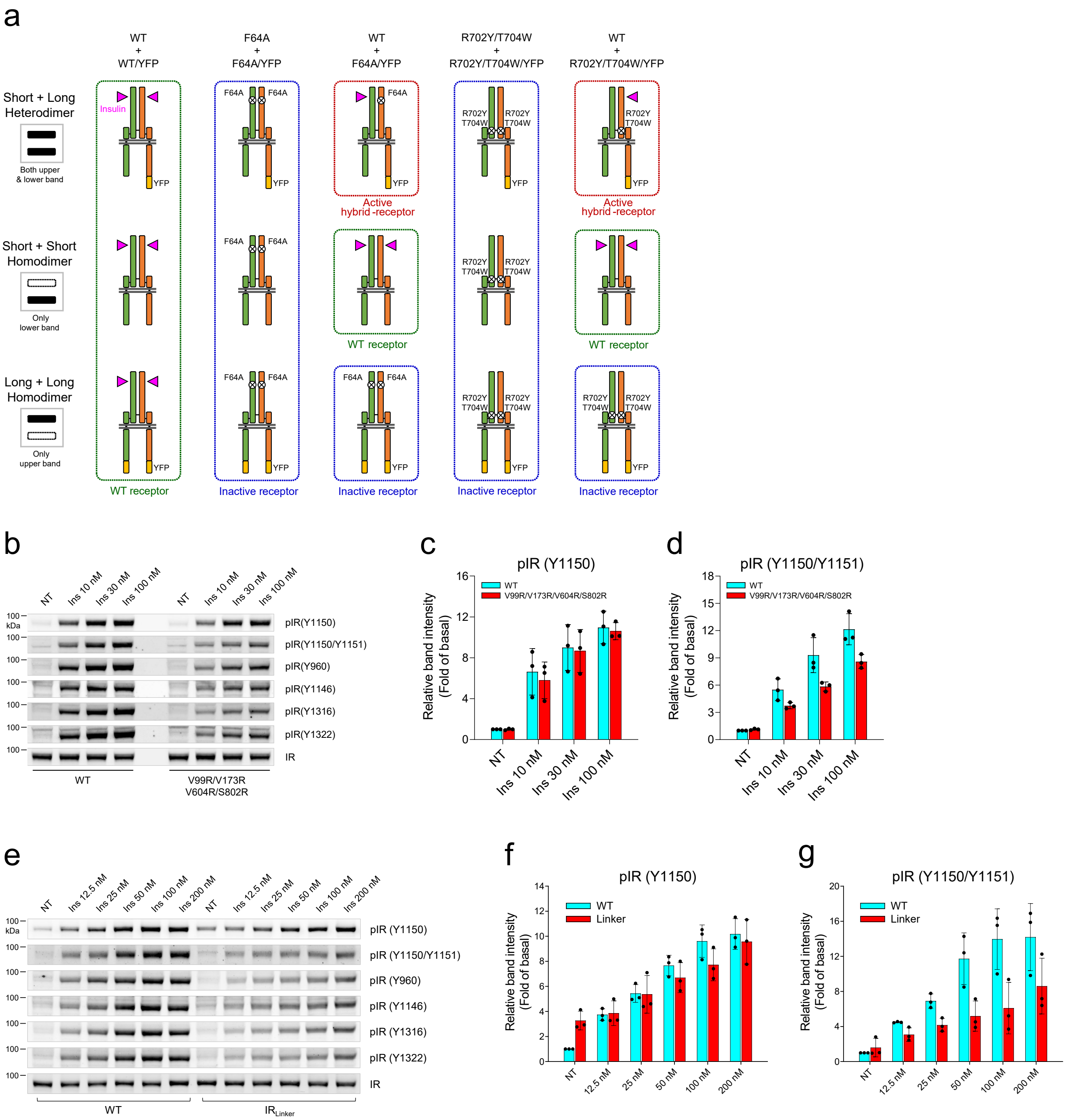

Supplementary Fig. 10. The position of the L1' domain modulates the transition of IR phosphorylation. a, Cartoon representation of all dimer combinations produced by co-transfection of short-IR (untagged) and long-IR (YFP-tagged). YFP-tagged long protomers are colored orange, and untagged short protomers are colored green. Insulin is colored pink, and YFP is colored yellow. The X circle symbols show the locations of mutations in each dimer combination. **b,** Insulin-induced IR phosphorylation in CHO-K1 cells expressing WT IR or the IR quadruple mutant (V99R, Val99 to Arg mutation; V173R, Val173 to Arg mutation; V604R, Val604 to Arg mutation; S802R, Ser802 to Arg mutation). Cells were stimulated with the indicated insulin concentrations for 5 min. The data were representative of three independent experiments. **c, d,** Quantification of western blot data for m-pY1150 or pY1150/pY1151 shown in Supplementary Fig. 10b. Experiments were repeated three times independently and graphs show means ± standard deviation (n=3). **e,** Insulin-induced IR phosphorylation in CHO-K1 cells expressing WT IR or the IR$_{linker}$ mutant. The data were representative of three independent experiments. **f, g,** Quantification of western blot data for m-pY1150 or pY1150/pY1151 shown in Supplementary Fig. 10e. Experiments were repeated three times independently and graphs show means ± standard deviation (n=3). Source data are provided as a Source Data file.

Supplementary Notes

Description of the structures of aptamers

Throughout this work, the entire naphthyl-modified deoxyuridine nucleotide (5-[N-(1-naphthylmethyl)carboxamide]-2'-deoxyuridine) is referred to as PX, the 2-napthyl moiety is 2NapX, and the uridine base is dUX (Supplementary Fig. 3a). The entire benzyl-modified deoxyuridine nucleotide (5-[N-benzylcarboxamide]-2'-deoxyuridine) is referred to as BX and the 5-benzyl moiety is 5BzX. The 2'-fluoro-modified bases are referred to as fX and 2'-O-methyl-modified bases are referred to as mX. X is the nucleotide number.

Description of the A62 structure

The A62 aptamer consists of 25 nucleotides in which seven dTs (deoxythymidine) are substituted by three Ps and four Bs (Supplementary Fig. 3a)[7]. A62 forms a non-helical compact structure, which is primarily stabilized through numerous base stacking interactions and Watson-Crick (WC) base pairs along with H-bonds and hydrophobic interactions (Fig. 2c).

At the lower part of A62, three successive WC base pairs (dA5-dB24, fC6-dG23, and dG7-fC22) are highlighted between the stem and the side loops (Fig. 2e, Supplementary Fig. 3c). Below the dA5-dB24 base pair, the dC1 to dB4 chain is positioned at the bottom with bases vertically oriented with respect to the dA5-dB24 bases. The 2Nap20 ring is sandwiched between the dA5-dB24 base pair and the dC1-dB4 chain, and the dU20 base is vertically oriented with respect to 2Nap20, forming a three-layer stack with B4(dU) and B24(Bz) at the back of 2Nap20 (Supplementary Fig. 3c).

In the middle region, the 2Nap10 ring is stacked on top of the dG7-fC22 base pairs on one side, and also reciprocally stacked on the dA9 base on another side (Fig. 2e). The mG11 base is sandwiched between the dU10 and fA19 bases, forming a four-layer stack, which is vertically oriented with respect to the three WC base pairs at the lower part, which forces a significant bend in the aptamer. On top, the dU14 base occupies the center of the head loop, forming a three-layer stack with the mG13 and dP16 (2Nap) bases (Fig. 2e, Supplementary Fig. 4f). The 5Bz14 resides on top of the four-layer stack (dA9-dU10-mG11-fA19) and is perpendicularly packed against another four-layer stack formed by dU16, dA17, dG18, and fA12 at the side. The three base-base stacking interactions (dU14, mG13, and 2Nap16) on top force the C15 base to flip, allowing it to interact with IR (R14 and F64 at L1). Overall, several modified bases engage in hydrophobic, H-bond, and stacking interactions to stabilize A62 and allow the aptamer to interact with IR.

Interaction between A62 and IR

Due to their internal stacking, several bases are flipped and exposed in both side loops and a head loop, enabling A62 to interact with L1 and FnIII-1' of IR (Fig. 2d–g). In the L1 site, loop H and L make contacts with L1-β2 (Fig. 2d, f). This interface is stabilized through extensive hydrophobic interactions, ion pairs, and H-bonds between A62 bases and IR residues. On the opposite side, a flat face is formed by the stem and two loops packed against the side of the main β-sheet of FnIII-1' through stacking between modified bases and β-sheet residues, and ion pairs between the A62 phosphate backbone and basic residues (Fig. 2d, g). In the FnIII-1'–loop S interface, three nucleotides (fA19, dP20, and mC21) bind to residues of FnIII-1', the dA19 base engages in hydrophobic interactions with L552, and phosphate groups of dP20 and mC21 interact with R488 and Q546, respectively. The L1–loop H interface involves docking of 2Nap16 into the surface pocket formed by F64, R65, F88, F89, F96, and R118 (Fig. 2f). Two residue–phosphate interactions (between dP16 and Tyr60 and Gln34, and between dA17 and R14) augment the interface (Fig. 2g). In the L1–loop L interface, the dA17 phosphate group binds to Y67, and the ribose ring of dA9 forms an H-bond with R65. In the FnIII-1'–stem interface, the dU3 base interacts with K544 and His548, and the dU4 base is reciprocally sandwiched between Y477 and dU20.

Description of the A43 structure

The A43 aptamer consists of 31 nucleotides in which six dTs are substituted by Ps (Supplementary Fig. 6a)[8]. The structure of the A43 aptamer is maintained primarily through numerous multilayer base-base stacking interactions highlighted by (i) dT4, dG5, dU6, dA7, and reciprocally 2Nap24; (ii) dG27, reciprocally 2Nap6, dC28, dG29, dA30, and dC31 (Supplementary Fig. 6c); (iii) dC18, dG19, dG20, dC21, dA22, and reciprocally dC9; (iv) dG11, dC12, dA13, reciprocally 2Nap17, and dA16; (Supplementary Fig. 6d). Non-canonical base pairs (dG5-dG29 and dA7-dG27) and a WC base pair (dT4-dA30) further stabilize the aptamer structure (Supplementary Fig. 6c).

Interactions between A43 and IR

The major groove packs against the main β-sheet of FnIII-1', whereas the shallow groove on the opposite side and the stem region are exposed to the surface without contacting IR (Fig 3d, e;

Supplementary Fig. 6c–f, h–j). The loop makes contacts with CR and L2, and it is located close to L1 and αCT' (Fig. 3d and Supplementary Fig. 6e, f, h–j). The A43-IR interface is formed from stacking interactions between residues and bases, including Tyr477-dC25, Trp551-dC10, and Arg271-dA16; electrostatic interactions between phosphate-basic residues (P24–Lys484; dG11–Ser323/T325; dC25–Arg479 and Tyr477; dC12–Asn348); and hydrophobic interactions (Supplementary Fig. 6e, f, h–j).

Four domains of IR interact with the A43 aptamer (Fig. 3d). First, L1 (R86) makes weak contacts to 2Nap15. Second, CR (R271, G273, C274, and H275) interacts with dP15 (2Nap), dA16, dP17(dU), and dC18 (ribose; Supplementary Fig. 6e). Third, L2 (S323 and T325) makes contact with the phosphate group of G11 (Supplementary Fig. 6f). Fourth, FnIII-1' engages in extensive interactions with various nucleotides, including residue–phosphate interactions (S481–dP24, R479–dC25, K544–dC28) and residue–base interactions (R488 and Q546–dU8, L486 and L552–2Nap8, W551–dC10, Q546–dU24, and Y477–dC25; Supplementary Fig. 6h–j).

Supplementary references

1. Croll, T. I. et al. Higher-Resolution Structure of the Human Insulin Receptor Ectodomain Multi-Modal Inclusion of the Insert Domain. *Structure (London, England : 1993)* **24**, 469-476 (2016).
2. Menting, J.G. et al. How insulin engages its primary binding site on the insulin receptor. *Nature* **493**, 241-5 (2013).
3. Weis, F. et al. The signalling conformation of the insulin receptor ectodomain. *Nature Communications* **9**, 4420 (2018).
4. Uchikawa, E. et al. Activation mechanism of the insulin receptor revealed by cryo-EM structure of the fully liganded receptor-ligand complex. *Elife* **8,** e48630 (2019).
5. Li, J. et al. Synergistic activation of the insulin receptor via two distinct sites. *Nature Structural& Molecular Biology* **29,** 357-368 (2022).
6. Gutmann, T. et al. Visualization of ligand-induced transmembrane signaling in the full-length human insulin receptor. *J Cell Biol* **217**, 1643-1649 (2018).
7. Yunn, N.-O. et al. An aptamer agonist of the insulin receptor acts as a positive or negative allosteric modulator, depending on its concentration. *Exp Mol Med* **54,** 531–541 (2022).
8. Yunn, N.-O. et al. A hotspot for enhancing insulin receptor activation revealed by a

conformation-specific allosteric aptamer. *Nucleic Acids Research* **49**, 700-712 (2021).

Supplementary Table 1 | Cryo-EM data collection, refinement and validation statistics.

	IR_{2xA62} (EMD-34021) (PDB 7YQ6)	[a]$IR_{A62+Ins}$ (EMD-34020) (PDB 7YQ5)	[a]$IR_{A62+Ins}$ (Upper part) (EMD-34019) (PDB 7YQ4)	[b]$IR_{A43+Ins}$ (EMD-34018) (PDB 7YQ3)	[b]IR_{2xIns} (EMD-34281) (PDB 8GUY)
Magnification	79,000	105,000		79,000	
Voltage (kV)	200	300		200	
Electron exposure (e–/Å^2)	50	50		50	
Defocus range (μm)	-1.5 to -3.0	-0.5 to -2.25		-1.5 to -3.0	
Pixel size (Å)	1.06995	0.85		1.06995	
Symmetry imposed	C2	C1	C1	C1	C2
Initial particle images (no.)	3,0611,20	2,614,086	2,614,086	5,089,489	5,089,489
Final particle images (no.)	181,797	163,150	163,150	375,895	127,787
Map resolution (Å)	4.18	4.27	3.95	3.62	
FSC threshold	0.143	0.143	0.143	0.143	0.143
Initial model used (PDB code)	$IR_{A62+Ins}$	$IR_{A43+Ins}$/4ZXB	$IR_{A43+Ins}$/4ZXB	6HN5/6HN4 /6PYH	6PXV
Model resolution (Å)	4.5	4.5	4.2	3.6	
FSC threshold	0.5	0.5	0.5	0.5	0.5
Map sharpening *B* factor (Å^2)	-195.5	-172.4	-131.1	-119.4	-190.8
Model composition					
Non-hydrogen atoms	13,990	14,067	10,618	14,412	14,222
Protein residues	1,592	1,673	1,243	1,707	1762
Nucleotide	48	24	24	28	0
B factors (Å^2)					
Protein	143.91	75.78	41.81	150.86	159.41
Nucleotide	54.75	24.97	24.16	160.73	
R.m.s. deviations					
Bond lengths (Å)	0.005	0.006	0.006	0.005	0.005
Bond angles (°)	1.120	1.126	1.158	1.052	0.997
Validation					
MolProbity score	1.91	1.82	1.74	1.81	1.88
Clashscore	7.50	5.39	4.50	5.48	6.07
Poor rotamers (%)	0.35	0.59	0.53	0.32	0.12
Ramachandran plot					
Favored (%)	91.52	90.69	91.05	91.11	90.05
Allowed (%)	8.48	9.31	8.95	8.89	9.95
Disallowed (%)	0	0	0	0	0

[a] Same data set was used for the $IR_{A62+Ins}$ complex

[b] Same data set was used for $IR_{A43+Ins}$ and IR_{2xIns}

Supplementary Table 2 | Reagent information

Reagent	Source	Identifier
**Antibodies**		
Anti-insulin receptor β	Santa cruz	Cat# sc-57342
Anti-phospho-insulin receptor β (Y1150)	Santa cruz	Cat# sc-81500
Anti-phospho-insulin receptor β (Y1150/Y1151)	Invitrogen	Cat# 44-804G
Anti-phospho-insulin receptor β (Y960)	Invitrogen	Cat# 44-800G
Anti-phospho-insulin receptor β (Y1146)	Cell signaling	Cat# 80732
Anti-phospho-insulin receptor β (Y1316)	Invitrogen	Cat# 44-807G
Anti-phospho-insulin receptor β (Y1322)	Invitrogen	Cat# 44-809G
Goat anti-rabbit IgG conjugated to DyLight 800	Invitrogen	Cat# SA5-35571
Goat anti-mouse IgG conjugated to DyLight 800	Invitrogen	Cat# SA5-35521
Goat anti-rabbit IgG conjugated to IRdye 680LT	Li-cor	Cat# 926-68021
Goat anti-mouse IgG conjugated to IRdye 680LT	Li-cor	Cat# 926-68020
**Chemicals, peptides, and recombinant proteins**		
Dulbecco's Modified Eagle's Medium, high glucose	Lonza	Cat# 12-604f
Opti-MEM I Reduced Serum Medium	Gibco	Cat# 31985-070
F-12 Nutrient Mixture Ham Kaighn's Modification	Welgen	Cat# LM 010-03
TrypLE™ Express (1X), Phenol Red	Gibco	Cat# 12605028
Lipofectamine® 3000 Transfection Reagent	Invitrogen	Cat# L3000-008
Antibiotic-Antimycotic	Gibco	Cat# 15240-062
Polyethylenimine	PolyScience	Cat# 23966
Geneticin™ Selective Antibiotic (G418 Sulfate)	Gibco	Cat# 11811031
HEPES	Sigma-Aldrich	Cat# H4034
Phosphate-buffered saline	Lonza	Cat# 17-517Q
Tris	Roche	Cat# 10708976001
Ethylenediaminetetraacetic acid (EDTA)	Sigma-Aldrich	Cat# E6758
Phenylmethanesulfonyl fluoride (PMSF)	Sigma-Aldrich	Cat# P7626
Sodium fluoride	Sigma-Aldrich	Cat# 201154
β-Glycerophosphate disodium salt	Chem Cruz	Cat# SC-220452A
Sodium orthovanadate	Sigma-Aldrich	Cat# S6508
Glycerol	Samchun	Cat# 170
Triton X-100	Alfa Aesar	Cat# A16046
Sodium dodecyl sulfate (SDS)	VWR Life science	Cat# 0227
Sodium deoxycholate	Sigma-Aldrich	Cat# D6750
Recombinant human Insulin	Sigma-Aldrich	Cat# 91077C
cOmplete™, EDTA-free Protease Inhibitor Cocktail	Sigma-Aldrich	Cat# 5056489001
n-Dodecyl-β-D-Maltopyranoside (DDM)	Anatrace	Cat# D310S
Cholesteryl hemisuccinate tris salt	Sigma-Aldrich	Cat# C6103
Gibco® FreeStyle™ 293 Expression Medium	ThermoFisher SCIENTIFIC	Cat# 12338026

FLAG-peptide	Anygen	Cat# AGP-8822
Fetal bovine serum (FBS)	Gibco	Cat# A3160402
**Experimental models: Cell lines**		
Rat-1 cell overexpressing human IR	UC San Diego	N/A
CHO-K1cell	ATCC	Cat# CCL-61
293-F Cell	Invitrogen	Cat# R79007
293-F Cell overexpressing human IR	This paper	N/A
**Oligonucleotides**		
IR-A62 aptamer	Aptamer science	Custom order
IR-A43 aptamer	Aptamer science	Custom order
**Recombinant DNA**		
Human IR-His-Flag (WT), pcDNA3.1	This paper	N/A
Human IR (F705A), pcDNA3.1	This paper	N/A
Human IR (R702Y/T704W), pcDNA3.1	This paper	N/A
Human IR (R14A), pcDNA3.1	This paper	N/A
Human IR (K40A), pcDNA3.1	This paper	N/A
Human IR (F64A), pcDNA3.1	This paper	N/A
Human IR (R65A), pcDNA3.1	This paper	N/A
Human IR (Y477A), pcDNA3.1	This paper	N/A
Human IR (R479A), pcDNA3.1	This paper	N/A
Human IR (R488A), pcDNA3.1	This paper	N/A
Human IR (R554A), pcDNA3.1	This paper	N/A
Human IR (R271A), pcDNA3.1	This paper	N/A
Human IR (Q272A), pcDNA3.1	This paper	N/A
Human IR (S323A), pcDNA3.1	This paper	N/A
Human IR (T325A), pcDNA3.1	This paper	N/A
Human IR (Y477A), pcDNA3.1	This paper	N/A
Human IR (K484A), pcDNA3.1	This paper	N/A
Human IR (L486A), pcDNA3.1	This paper	N/A
Human IR (R488A), pcDNA3.1	This paper	N/A
Human IR (W551A), pcDNA3.1	This paper	N/A
Human IR (L552A), pcDNA3.1	This paper	N/A
Human IR (R554A), pcDNA3.1	This paper	N/A
Human IR (WT), pcDNA3.1	This paper	N/A
Human IR-YFP (WT), pcDNA3.1	This paper	N/A
Human IR (F64A), pcDNA3.1	This paper	N/A
Human IR-YFP (F64A), pcDNA3.1	This paper	N/A
Human IR (R702Y/T704), pcDNA3.1	This paper	N/A
Human IR-YFP (R702Y/T704), pcDNA3.1	This paper	N/A
Human IR (V99R/ V173R/V604R/S802R), pcDNA3.1	This paper	N/A
Human IR (Linker), pcDNA3.1	This paper	N/A
Human IR (Y972A), pcDNA3.1	This paper	N/A
**Other**		

Superose 6 10/300 increase	GE Healthcare	Cat# 29-0915-96
30 kDa MWCO concentrator	Amicon Ultra, Merck	Cat# UFC910024
Vivaspin® 500, 100 kDa MWCO	GE Healthcare	Cat# 28932237
Anti-DYKDDDDK G1 Affinity Resin	Genscript	Cat# L00432
C-flat 1.2/1.3 Au 400mesh	EMS	Cat# CF413-50-Au
